# *De novo* assembly and functional annotation of blood transcriptome of loggerhead turtle, and *in silico* characterization of peroxiredoxins and thioredoxins

**DOI:** 10.7717/peerj.12395

**Published:** 2021-11-18

**Authors:** Javier Hernández-Fernández, Andrés Mauricio Pinzón Velasco, Ellie Anne López Barrera, María Del Pilar Rodríguez Becerra, José Luis Villanueva-Cañas, M. Mar Alba, Leonardo Mariño Ramírez

**Affiliations:** 1 Department of Natural and Environmental Sciences, Faculty of Science and Engineering, Genetics, Molecular Biology and Bioinformatic Research Group—GENBIMOL, Universidad Jorge Tadeo Lozano, Bogotá, D.C., Colombia; 2 Faculty of Sciences, Department of Biology, Pontificia Universidad Javeriana, Bogotá, D.C., Colombia; 3 Grupo de Bioinformática y Biología de Sistemas, Universidad Nacional de Colombia, Bogotá, Colombia; 4 Institute of Environmental Studies and Services. IDEASA Research Group—IDEASA, Sergio Arboleda University, Bogotá, D.C., Colombia; 5 Molecular Biology CORE (CDB), Hospital Clínic de Barcelona, Barcelona, Spain; 6 Evolutionary Genomics Group, Research Program on Biomedical Informatics (GRIB), Hospital del Mar Research Institute (IMIM), Universitat Pompeu Fabra, Barcelona, Spain; 7 Catalan Institution for Research and Advanced Studies (ICREA), Barcelona, Spain; 8 Computational Biology Branch, NCBI, NLM, NIH, Bethesda, MD, United States of America

**Keywords:** *Caretta caretta*, Transcriptome, Peroxiredoxin, Thioredoxin, KEGG pathway, Blood, RNA-seq, 3D modelling

## Abstract

The aim of this study was to generate and analyze the atlas of the loggerhead turtle blood transcriptome by RNA-seq, as well as identify and characterize thioredoxin (Tnxs) and peroxiredoxin (Prdxs) antioxidant enzymes of the greatest interest in the control of peroxide levels and other biological functions. The transcriptome of loggerhead turtle was sequenced using the Illumina Hiseq 2000 platform and *de novo* assembly was performed using the Trinity pipeline. The assembly comprised 515,597 contigs with an N50 of 2,631 bp. Contigs were analyzed with CD-Hit obtaining 374,545 unigenes, of which 165,676 had ORFs encoding putative proteins longer than 100 amino acids. A total of 52,147 (31.5%) of these transcripts had significant homology matches in at least one of the five databases used. From the enrichment of GO terms, 180 proteins with antioxidant activity were identified, among these 28 Prdxs and 50 putative Tnxs. The putative proteins of loggerhead turtles encoded by the genes Prdx1, Prdx3, Prdx5, Prdx6, Txn and Txnip were predicted and characterized *in silico*. When comparing Prdxs and Txns of loggerhead turtle with homologous human proteins, they showed 18 (9%), 52 (18%) 94 (43%), 36 (16%), 35 (33%) and 74 (19%) amino acid mutations respectively. However, they showed high conservation in active sites and structural motifs (98%), with few specific modifications. Of these, Prdx1, Prdx3, Prdx5, Prdx6, Txn and Txnip presented 0, 25, 18, three, six and two deleterious changes. This study provides a high quality blood transcriptome and functional annotation of loggerhead sea turtles.

## Introduction

The industrial development and exploitation of coastal regions are associated with the direct or indirect introduction of anthropogenic substances or energy to the marine ecosystem, producing high pollution, and therefore, harmful effects on animal and plant life (D’ilio et al., 2011; Gallen et al., 2019). Stressors resulting from contamination are associated with toxicity and bio-accumulation of substances in living organisms, including sea turtles (Da Silva & López-Barrera, 2016). Other appearing phenomena such as global warming, salinization, and acidification of the oceans can increase xenobiotics bioavailability, affect marine ecosystems, and generate contamination risk in a rapidly changing environment (Schiedek et al., 2007). Their effects on life are not easily quantifiable, a situation that does not allow studying the significance of all these factors and their influence on populations and their habitats.

Sea turtles have been used as indicators or sentinels of environmental pollution, recording the health status of coastal and marine environments both locally and globally (Eckert et al., 2000; Heithaus, 2013). These species play an important role in the transport of pollutants between the different trophic networks, which could currently be underestimated due to lack of information (Gardner, 2016).

The loggerhead sea turtle, *Caretta caretta*, is a highly migratory sea turtle species with a global distribution. Its migratory behavior coupled with its position in the food chain make this species most suitable as a sentinel of environmental contamination. As an omnivore, the loggerhead sea turtle is the sea turtle species with the most varied diet: sponges, corals, sea feathers, polychaete worms, sea anemones, barnacles, cephalopods, brachiopods, isopods, and even insects (Duermit, 2007; Hernández-Fernández et al., 2020). This species is an important component of complex marine and coastal ecological systems (Eckert et al., 2000) because it occupies a wide diversity of habitats during its life cycle and maintains a balance in the ecosystems by controlling prey populations (Otálora & Hernández-Fernández, 2018). The loggerhead sea turtle has been detected in the Colombian Caribbean. Nesting beaches have been identified in the regions (departments) of Magdalena, Guajira, Bolívar, and San Andrés (INVEMAR, 2003; Ceballos-Fonseca, 2004).

The loggerhead turtle is considered globally vulnerable under the current criteria of the IUCN Red List (criterion A2b) (IUCN-2020-1) (Casale & Tucker, 2017) and is critically endangered (CR A2cd) under the red reptile book for Colombia (Morales-Betancourt, Páez y & Lasso, 2015). Nesting female populations have dropped considerably from 600 females arriving in the Colombian Caribbean in 1979 to just 6–10 today (Moreno Munar & Marino, 2017; Ramìrez-Gallego & Barrientos-Muñoz, 2020). Amaya-Espinel & Zapata (2014) estimate this species is practically extinct in Colombia, and the few females that still nest only do so between the Palomino River (La Guajira) and the Piedras River (Magdalena).

For the aforementioned reasons, the identification of populations especially vulnerable to interactions and responses to pollution is a pressing issue, especially in the context of the many physical, chemical and biological stressors that will be enhanced by climate change. Stressors or environmental pollutants generate oxidative stress and constantly affect the internal equilibrium of cells in turtles, altering the levels of antioxidant enzymes that act as a barrier to protect the body’s cells (Hernández-Fernández et al., 2020; Finlayson, Leusch & van de Merwe, 2018). The development of genomics (study of how the genome, the complete set of genes and their products, is translated into biological functions) provides tools that can help us understand how widespread contamination can affect the health of turtles and the ecosystem (Snape et al., 2004).

RNA-Seq, using Next-Generation Sequencing (NGS), is now the most widely used technology for obtaining the transcriptome of a species (Söllner et al., 2017; Geniza & Jaiswal, 2017; Fox et al., 2014) and scrutinizing genes that are actively transcribed at a given stage of its life. The numerous bioinformatics programs used to assemble a *de novo* transcriptome have allowed the study of non-model organisms, for which there is no sequenced reference genome, such as the loggerhead turtle. A *de novo* transcriptome assembly can be used to explore genetic diversity, extract potential genetic markers, and study and elucidate different physiological and pathological factors in animals (Fox et al., 2014; Hernández-Fernández, Pinzón & Mariño Ramírez, 2017). However, studies of the latter have not been carried out on sea turtles. Recently, Bentley et al. (2017) obtained a *de novo* transcriptome of the brain of developing loggerhead embryos to study embryonic responses to heat stress during incubation. The results confirmed that genes of the Hsp family play an important role in tolerance to heat stress during development and, in parallel with other genes, they must be evaluated to understand the capacity of oviparous reptiles to respond to the effects of climate change.

Prior to the current, Hernández-Fernández, Pinzón & Mariño Ramírez (2017) presented a reduced version of the loggerhead turtle transcriptome. Similarly, Hernández-Fernández et al. (2021) investigated the changes in enzyme activity and gene expression of erythrocytes from exposure of the loggerhead turtle to methylmercury, RNAseq was used to assemble the *de novo* transcriptome showing that methylmercury altered gene expression patterns in response to the cellular stress produced. These reflected in lysosomal activity, mitochondrial regulation, cell cycle regulation, autophagy, calcium regulation, apoptosis, regulation of transcription and translation. Hernández-Fernández et al. (2021) substantiates that different stressors produced by environmental pollution can trigger a response against oxidative stress in sea turtles (Da Silva & Lopez-Barrera, 2016).

On the other hand, several RNA-seq studies have been carried out with the *Chelonia mydas* sea turtle to elucidate the molecular mechanisms involved in fibropapillomatosis (Duffy et al., 2018; Yetsko et al., 2021; Blackburn et al., 2021). Duffy et al. (2018) performed the assembly of *de novo* transcriptome from biopsies of green turtles with fibropapillomatosis, revealing the signaling pathways involved in tumors. Yetsko et al. (2021) obtained the transcriptome of *Chelonia mydas* individuals with internal and external tumors and found that internal tumors are transcriptionally different from external ones. Blackburn et al. (2021) obtained the transcriptome of green turtles with and without fibropapillomatosis and found that blockage in the wnt pathway is the main characteristic of tumors produced by fibropapilloma.

Loggerhead turtles possess a powerful antioxidant defense system that protects them from reactive oxygen species (ROS) that are constantly generated inside and outside the cell by oxidation–reduction reactions (Hernández-Fernández et al., 2020; Bentley et al., 2017). This system is composed of the enzymes SOD, CAT, GST, Tnxs and Prdxs among others, which maintain cellular homeostasis (Franco, Navarro & Martínez-Pinilla, 2019). Of all these enzymes, thioredoxins and peroxiredoxins have received little attention in their expression in turtles (Zhang et al., 2019). Studies conducted on these reptiles have focused on assessing how brevetoxins affect Tnxs, GST, and SOD levels (Walsh et al., 2019) and how turtles regulate intracellular levels of GST and Prdxs during oxidative stress caused by anoxia, freezing, and exposure to lipopolysaccharides (Krivoruchko & Storey, 2010; Storey, 2006; Xu et al., 2016). The Prdx and Tnxs families are ubiquitous cysteine dependents. They are found in all tissues fulfilling various functions related to cell survival and maintenance, protein repair, cell cycle regulation, defense against oxidative stress, modulation of the immune response and, very importantly, the control of the levels of hydrogen peroxides, lipid hydroperoxides, peroxynitrite and also in signal transduction using H_2_O_2_ as a second messenger (Nelson et al., 2018; Rhee, 2016; Hall, Karplus & Poole, 2009; Perkins et al., 2015). Thioredoxins are essential components of the Tnx system characterized by a conserved Trp-Cys-Gly-Pro-Cys-Lys sequence at its active site (Zhang et al., 2017). Structurally and functionally, Tnxs and Prdxs have been extensively studied in mammals and fish (Zhang et al., 2017; Zhang et al., 2019; Nadarajapillai et al., 2020; Shahnaj et al., 2020) and their roles in diseases such as cancer, Alzheimer’s, heart attack and bacterial infections have been investigated. In reptiles, especially turtles, these important protein classes remain uncharacterized.

We generated a high quality *de novo* transcriptome assembly from total RNA extracted from blood tissue in hatchlings, juveniles, and adults in captive conditions. The three main objectives of this study were: (1) to generate an atlas of the loggerhead turtle blood transcriptome genes that represent a comprehensive reference for further insight in molecular studies of this ecologically important species. (2) identify genes ontologically related to oxidative stress and (3) identify and characterize among them the thioredoxins and peroxiredoxins Prdx1, Prdx3, Prdx5, Prdx6, Txn, and Txnip, antioxidant enzymes of the greatest interest in the control of peroxide levels and other biological functions.

## Materials and Methods

### Ethical terms

The collection of biological samples and all methods used in this study follow the ethical standards established by the legislation and the ethics committee of the Jorge Tadeo Lozano University (Project 340-07-10). Samples were obtained under a research permit that was granted by the Ministry of Environment and Territorial Development (# 24 of June 22, 2012) and Contract for Access to Genetic Resources (# 64 of 129 April 23, 2013). A permit for the collection of samples of Colombian biodiversity, issued to the Jorge Tadeo Lozano University, was obtained for this study (Resolution 1271 of October 23, 2014, IDB040I File).

### Study area, sample collection, RNA extraction and analysis

Peripheral blood samples were collected from eight, healthy, captive-reared loggerhead sea turtles; three hatchling, three juveniles, and two adults. Additionally, we measured weight and carapace length and width of each turtle. The turtles were maintained at ambient temperature (average 30 °C, minimum 27 °C) in a natural outdoor seawater pool at the CEINER Oceanarium in San Martín de Pajares Island, Cartagena, Colombia (10°11′N, 75°47′W), (Fig. S1). Turtles were visually inspected for overall health and for any injuries to the flippers and carapace. Blood samples were extracted from the dorsal region of the cervical sinus, in accordance with Dutton (1996). For the collection of peripheral blood were used 1-,4- and 10 ml sterile syringes and sterile tubes with RNAlater^®^ solution (Ambion, Inc., Austin, TX, USA), (ratio 5:1 v/v), which prevents RNA degradation. We obtained 200 µl, 2 and 5 ml of blood from hatchling, juvenile and adult turtles respectively. Hatchling turtle samples were obtained from twenty-day old individuals and the adult and juvenile samples were obtained from foraging individuals in Islas del Rosario that had been kept in captivity. We used the three age classes to have a sample size as large as possible. The samples were transported at 4 °C to the Molecular Biology laboratory at the Jorge Tadeo Lozano University following the specifications of the manufacturer. Total RNA was extracted from all blood samples (*n* = 8) using the RNeasy^®^ Mini Kit (Qiagen, Hilden, Germany) following the specifications of the manufacturer. RNA integrity was inspected following a 1% agarose gel electrophoresis. Next, the RNA concentration was determined with the NanoDrop 1000 Spectrophotometer kit (Thermo Scientific, Denver, USA). The RIN and the rRNA ratio were evaluated using the Agilent 2100 BioAnalyzer (Agilent Technologies, CA, USA). Samples with RIN between 6-9,5 and an average of 7,5 were used to construct the libraries.

### Construction of mRNA libraries and sequencing

Complementary DNA was synthesized, and preparation of the cDNA libraries was performed with the TruSeq RNA simple Prep kit v2 following manufacturer instructions (Illumina, San Diego, CA.). The kit produces highly sensitive chain-specific libraries and selects RNA with a monophosphate group at the 5′ end and a hydroxyl group at the 3′ end, decreasing the error range. Total RNA (0.5 µg) was used for enrichment in polyA mRNA by oligo-dT magnetic beads, followed by fragmentation by divalent cations at high temperature, producing fragments of 80–250 nt. Each of the eight libraries were sequenced using the Illumina HiSeq-2000 platform in paired-end 100-nucleotide reads using TruSeq SBS Kit v3 (Macrogen, Korea). The schematic overview of the project is shown in Fig. S2.

### Quality control and filtering

We evaluated the raw sequencing data using the FastQC tool (Andrews, 2018). We used Trimmomatic (Bolger, Lohse & Usadel, 2014) to trim adapters, using the following parameters and values: PE, ILLUMINACLIP:adapters.fa:2:30:12:1:true, LEADING:3, TRAILING:3, MAXINFO:50:0.999, and MINLEN:75.

### Transcriptome assembly and quality evaluation

The *de novo* assembly of the loggerhead turtle transcriptome was generated with Trinity 2.8.5 (Haas et al., 2013), combining the reads of all 8 individuals. Subsequently, highly redundant contigs were removed with CD-Hit-EST v. 4.6.4 (Fu et al., 2012), using 0.95 as the identity parameter. After filtering, we obtained 374,545 unigenes. We evaluated quality of our *de novo* transcriptome using Transrate v1.0.3 (Smith-Unna et al., 2016) based on the statistics of the reconstructed transcripts and the read mappings. Additionally, we also assessed quality with the DOGMA V-3.4 tool (Kemena, Dohmen & Bornberg-Bauer, 2019), available at: https://domainworld.uni-muenster.de/programs/dogma/. This program measures the completeness of a given transcriptome or proteome based on a core set of conserved domain arrangements (CDAs) (Dohmen et al., 2016), in particular from Pfam (PfamScan v1.5; Pfam v32.0). Then, the open reading frames (ORFs) in each contig, and the corresponding proteins, were determined with the TransDecoder program, version 5.5.0 (Haas & Papanicolaou, 2016) (https://github.com/TransDecoder/TransDecoder/releases).

We also compared the assembled transcripts with the three available reference proteomes of other turtle species, and included humans using BLASTX (Altschul et al., 1997). The proteomes corresponded to the green sea turtle, *Chelonia Mydas* (NCBI, genebuild Mar-2014, GCA_000241765.2, Proteome ID UP000031443), western painted turtle, *Chrysemys picta bellii* (Ensembl, genebuild Dec-2018, GCA_000241765.2), Chinese soft-shelled turtle, *Pelodiscus sinensis* (Ensembl, genebuild Feb-2014, GCA_000230535.1, Proteome ID UP000007267) and humans, *Homo sapiens* (Ensembl, genebuild Mar-2019, GCA_000001405.27, Proteome ID UP000005640). These proteomes contained 28,672, 39,084, 20,669, and 109,914 protein sequences, respectively. The e-value cutoff was set to 1e^−10^.

### Functional annotation

The identified unigenes of the loggerhead turtle transcriptome, along with the ORFs extracted with TransDecoder, were annotated against the Uniprot-Sprot protein database (Jun 17, 2020) (UniProt, 2019) (https://www.uniprot.org/) using the Blastx/Blastp tools (*E*-value 1e^−5^). Additionally, the ORFs were annotated to the Pfam-A databases (May 1, 2020) (El-Gebali et al., 2019) (http://pfam.xfam.org/), EggNOG orthology (Huerta-Cepas et al., 2019), GO and KEEG database (35 organisms including 12 reptiles), using the online tools Blastp, EggNOG Mapper V 5.0 (http://eggnog5.embl.de/#/app/home) (Huerta-Cepas et al., 2017), and KEGG Automatic Annotation Server (KAAS) (http: //www.genome.jp/tools/kaas/) (Moriya et al., 2007).

The ontological terms (GO terms) obtained for each of the ORFs with EgNOG Mapper were then assigned to biological processes, molecular function, and cellular component using Web Gene Ontology Annotation Plot (WEGO) (Ye et al., 2018).

Annotations were plotted with the PlotsR tool designed for Rwizard 3.6 (Guisande, 2016)

### Identification characterization *in silico* of oxidative stress proteins

Putative antioxidant proteins, peroxiredoxins, and thioredoxins were identified based on the annotations made to the ORFs against the Gene Ontology database contained in eggNOG-mapper v2 (Huerta-Cepas et al., 2017), and with the WEGO tool. The amino acid and nucleotide sequences of each of the ORFs were extracted and their identities were corroborated by sequence similarity searches with the Blastx and Blastp tools (Altschul et al., 1997) against the nr database (https://blast.ncbi.nlm.nih.gov/Blast.cgi). The structural and functional characteristics of the predicted Prdx1, Prdx3, Prdx5, Prdx6, Txn and Txnip were analyzed. Length and molecular weight were determined with the EMBOSS_pepstat algorithm (https://www.ebi.ac.uk/Tools/seqstats/emboss_pepstats/). We inferred the functional domains by homology with the R-PS-Blast program and the CDD_Pfam V31.0 16709 PSSMs database (https://www.ncbi.nlm.nih.gov/Structure/cdd/docs/cdd_search.html). The motifs, active sites and their respective modifications were identified manually by performing a multiple alignment using Clustal Omega (https://www.ebi.ac.uk/Tools/msa/clustalo/) against the sequences of these same proteins present in model organisms and related species.

### Maximum Parsimony analysis between Prdxs, Txn and Txnip against other turtles

The coding sequences of genes Prdx1, Prdx3, Prdx5, Prdx6, Txn and Txnip conserved among the green sea turtle (*Chelonia mydas*), the western painted turtle (*Chrysemys picta bellii*), the soft-shell turtle (*Pelodiscus sinensis*) and loggerhead sea turtles (*Caretta caretta*), were extracted and aligned with guidance from amino-acid alignments created by the MUSCLE program (EBI Web Services). The sequences of the same proteins of humans (*Homo sapiens*) were used as outgroup for phylogenetic comparison. The phylogenetic tree showing the relationship between the proteins Prxd1, Prxd3, Prxd5, Prxd6, Txn and Txnip was inferred using the Maximum Parsimony method (MP). The bootstrap consensus tree inferred from 1,000 replicates is taken to represent the evolutionary history of the taxa analyzed (Nei & Kumar, 2000), with branches corresponding to partitions reproduced in less than 50% bootstrap replicates are collapsed. The percentage of replicate trees in which the associated taxa clustered together in the bootstrap test (1,000 replicates) are shown next to the branches (Nei & Kumar, 2000). The MP tree was obtained using the Subtree-Pruning-Regrafting (SPR) algorithm with search level 1 in which the initial trees were obtained by the random addition of sequences (10 replicates). This analysis involved 40 amino acid sequences, producing a total of 398 positions in the final dataset. The phylogenetic relationship analyses were conducted in MEGA X (Kumar et al., 2018).

### Identification of amino acid substitutions in predicted proteins

We predicted the amino acid changes found in the putative proteins Prdx1, Prdx3, Prdx5, Prdx6, Txn and Txnip of loggerhead sea turtles with respect to human proteins, and their possible impact on protein functionality. We used the SIFT program (Sorting Intolerant from Tolerant) (Ng & Henikoff, 2003) to analyze the function of an amino acid substitution because SIFT sorts intolerant from tolerant substitutions, and further classifies substitutions as tolerated or deleterious. SIFT proposes the hypothesis: “important positions in a protein sequence are conserved during evolution and, substitutions in these positions, affect protein function” (Sim et al., 2012, pg 452). Therefore, by using sequence homology, SIFT predicts the effects of all possible substitutions at each position in the protein sequence. SITF is available at: https://sift.bii.a-star.edu.sg/www/SIFT_seq_submit2.html.

### Prediction of 2D and 3D structures by homology

The complete sequences of the Prdxs and Txns that presented the greatest amount of amino acid changes, especially in the active site or in the structural motif, were modeled in 2D and 3D using the SOPMA (Geourjon & Deléage, 1995) and CPHmodels-3.2 server (Nielsen et al., 2010) programs respectively. These structures were compared with the homologues 2D and 3D human proteins.

### Physicochemical characterization of inferred proteins

The physicochemical characteristics of the predicted proteins that presented the greatest number of amino acid changes with respect to the proteins reported in humans were analyzed using the ProtParam tool available within the ExPASy server (Gasteiger et al., 2005).

### Data availability

The RNA sequencing reads in fastq format generated in the study have been deposited in the SRA NCBI database (Bioproject PRJNA560561, SAMN06350885, SAMN12591161, SAMN12591162, SAMN12591163, SAMN12591164, SAMN12591165, SAMN12591166, SAMN12591173 Sequence Read Archive: SRR10032986, SRR10032987, SRR10032988, SRR10032989, SRR10032990, SRR10032991, SRR10032992, SRR5330501, SRR10032986, SRR10032987, SRR10032988, SRR10032989, SRR10032990, SRR10032991, SRR10032992 and SRR5330501). The transcriptome assembly has been deposited at TSA (GenBank) under the accession GIBB00000000. The version described in this paper is the first version.

The sequences of genes and proteins predicted in this study were submitted to Genbank with the following GenBank accession numbers: MT833868 (Prdx1), MT833869 (Prdx3), MT833870 (Prdx5), MT833871 (Prdx6), MT833872 (Txn), and MT833873 (Txnip).

## Results

### Blood transcriptome sequencing and *de novo* assembly

We assembled total RNA from peripheral blood from eight loggerhead turtles (Table S1). The sequencing yielded 75.4 Gb of raw data from the loggerhead turtle transcriptome of various age classes and sizes. We remove adapters and sequences with low quality, eliminating 2.45% of the reads.

We used the filtered sequencing reads of the eight samples to perform a *de novo* transcript assembly. The complete assembly contained 515,597 individual contigs (transcripts) with sizes ranging from 200 to 29,898 bp, large genomes regularly produce equally large transcriptomes.

The N50 value is commonly used to evaluate an assembly (Bryant et al., 2017) and in this transcriptome, the N50 had a length of 2,631 bp. This means that at least half of the assembled bases are in contigs of that length (Table 1). This N50 value is similar to that previously reported for loggerhead sea turtles 2,585 bp (Bentley et al., 2017) and 3,520 bp (Hernández-Fernández et al., 2021). Our N50 value is much greater than the average contig length, suggesting that the transcriptome is of good quality and depth (Montero-Mendieta et al., 2017; Simpson, 2014). The GC content for the assembly was 46%, the median contig length was 998.74 bp, and the average contig length was 1,826 bp (Table 1). Bentley et al. (2017) reported for loggerhead turtle the same GC content of 46%, but much lower median contig length (415) and average contig length (1076.23).

**Table 1:** Contig metrics of transcriptome of *C. caretta*.

**Parameter**	**Trinity statistics**
**Total contigs (transcripts)**	515,597
**Median contig length**	998,74
**Average contig length**	1826
**N30**	4579
**N50**	2631
**N70**	974
**N90**	324
**GC content**	0.46

To perform a first assessment of the protein coding gene content of the newly generated transcriptome (515,597 transcripts), we ran BLASTX against the proteomes of three other Testudines species, and humans as outgroup. From the BLASTX results we identified 17,570 unique proteins in the green sea turtle (*Chelonia mydas*), 22,079 in the western painted turtle (*Chrysemys picta bellii*), 14,714 in the soft-shell turtle (*Pelodiscus sinensis*) and 23,607 in humans (*Homo sapiens*). We think that the differences between the numbers of hits across species most likely reflect the different degree of completeness of the protein annotations in these species. Our analysis indicated that the transcriptome of the loggerhead sea turtle contains many of the genes identified in other species.

We also evaluated the quality of the *de novo* transcriptome with Transrate v1.0.3 (Smith-Unna et al., 2016). This tool can easily detect multiple common artifacts of the *de novo* assembly, including chimeras, structural errors, incomplete assembly, and base calling errors. Most transcripts, 376,561 (64%), were identified as good quality by Transrate. Additionally, we used DOGMA to assess the level of completeness of the transcriptome and identified 76.4% and 88.6% of the vertebrate and eukaryotic core sets, respectively. Taken together, these results support high quality and completeness of our transcriptome of the loggerhead turtle.

### Protein coding transcripts

Complete transcriptome assemblies typically include highly redundant transcripts, including polymorphic variants and lowly expressed isoforms that represent significant challenges for downstream analysis of the proteins (Sayadi et al., 2016). For this reason, our transcriptome was further processed with CD-Hit-EST (Fu et al., 2012), a hierarchical grouping tool that allows the fusion of assembled transcripts that share high identity (Fu et al., 2012; Chen et al., 2017). This reduced the initial set of 515,597 transcripts to 374,545 unigenes with at most 95% identity between them.

Trinity generates duplicate contigs as it cannot distinguish similar transcripts from each other, causing a large number of redundant contigs (Hsieh, Oyang & Chen, 2019; Bushmanova et al., 2019).

We identified 165,676 open reading frames (ORFs) likely to represent bona fide coding sequences. CDs had between 300 and 22,617 bp (Fig. S3), with an average of 3,803 bp. 55.6% of the CDs (92,186) were considered to be complete.

### Annotation of protein-coding transcripts

Transcripts likely to encode proteins underwent further downstream analyses (165,676 ORF). We found that 27,823 of them had significant sequence similarity to Uniprot_Sprot proteins (28,209 against Uniprot). We performed searches against Pfam and EggNOG and found that a total of 52,147 genes showed significant similarity with proteins in at least one database (Table 2). This number of identified protein-coding genes is consistent with that described for other turtles (Wang et al., 2013; Yin et al., 2016). Of the 37 assembled turtle genomes (Genbank – 369 Genomes – NCBI Datasets), only 10 have been published. In the TSA (Transcriptome Shotgun Assembly Database) only the transcriptome submitted by this study appears as the master record for transcriptome assembly projects (accession GIBB00000000.1).

**Table 2:** Database distribution of annotated unigenes.

**Values**	**Total**	**Uniprot_Sprot Blastp**	**Uniprot_Sprot Blastx**	**Pfam** **Blastp**	**KEGG**	**EggNOG**	**GO**	**Partial intersection**	**Overall**
Number unigenes	165,676	27,823	28,209	44,587	17,046	49,612	39,421	8,663	52,147
Percentage	100%	16.79%	17%	26.91%	10.28%	29.90%	23.79%	5.22%	31.47%

### Functional analysis with Gene Ontology

**Figure 1:**
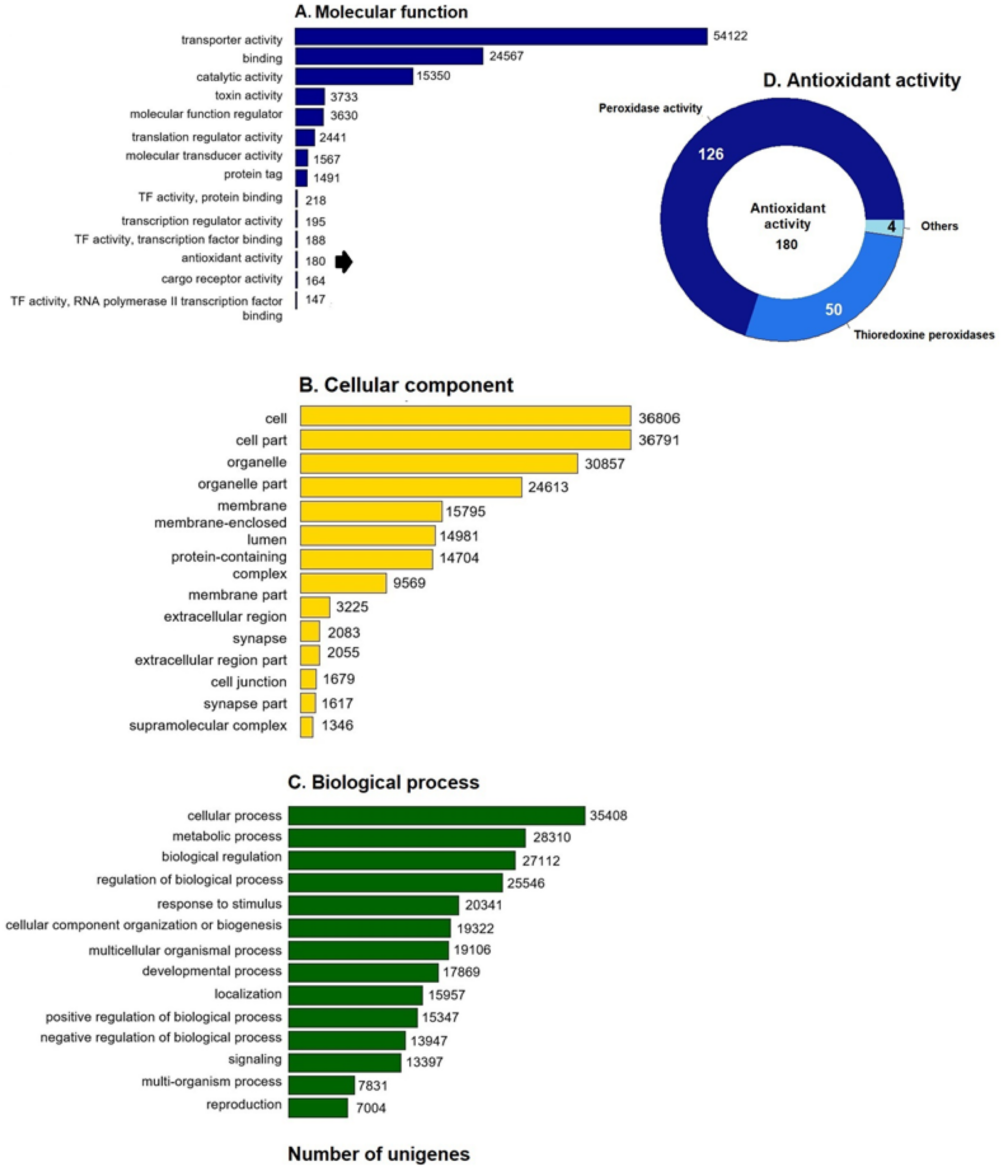
GO unigenes classification of loggerhead turtle. Distribution of the 14 main sub-categories (Level 2) of the GO unigenes classification of loggerhead turtle using WEGO tool. The obtained unigenes were classified into three GO categories: (A) molecular function, (B) biological process, (C) cellular component. (D) Number of enzymes with antioxidant activity identified.

GO enrichment analysis of the putative loggerhead turtle proteins was performed, in total, 107,175 GO terms originating from 76 functional groups (GO level 2) were assigned to 39,421 unigenes (Fig. 1). The analysis identified a multitude of proteins with a putative regulatory function (27,112) or associated to organelles (24,613). Among the most representative subcategories of molecular function were transport activity, binding activity, and catalytic activity in which 54,122 (50.5%), 24,567 (22.9%) and 15,350 (14.3%) terms were assigned, respectively (Fig. 1A). Additionally, 180 protein sequences related to antioxidant activity were identified, out of which 126 presented peroxidase activity, 27 peroxiredoxins, 23 with thioredoxin-disulfide reductase activity, 50 thioredoxin peroxidases, and 4, 3 and 2 with superoxide dismutase activity, sulfiredoxin activity, and glutathione dehydrogenase, respectively (Fig. 1D).

### Protein Domain Identification

Prediction of functional domains is a key step in transcriptome annotation because it provides basic information on cellular functions of domain families. Pfam identified 44,587 putative proteins with conserved domains (Table 2). The most populated categories were the following: 830 protein kinase domains (1.86%), 593 7-transmembrane-receptor domains (rhodopsin family) (1.33%), 593 leucine rich repeat domains (1.33%), 545 WD domain G-beta repeat (1.2%), 442 Immunoglobulin V-set domains (0.99%), 435 zinc finger C2H2-type domains (0.97%), 395 protein tyrosine kinase domains (0.88%), 348 reverse transcriptase (RNA-dependent DNA polymerase) domains (0.78%), 332 PDZ domains (0.74%), and 324 spectrin repeat domains (0.72%).

The WD domain G-beta repeat was the fourth most common Pfam domain. These proteins are relevant in critical roles in many biological functions, such as signal translation, regulation of transcription, and apoptosis (Umbarger et al., 1992). These proteins have not been studied in turtles yet.

### Orthologous genes diversity

**Figure 2:**
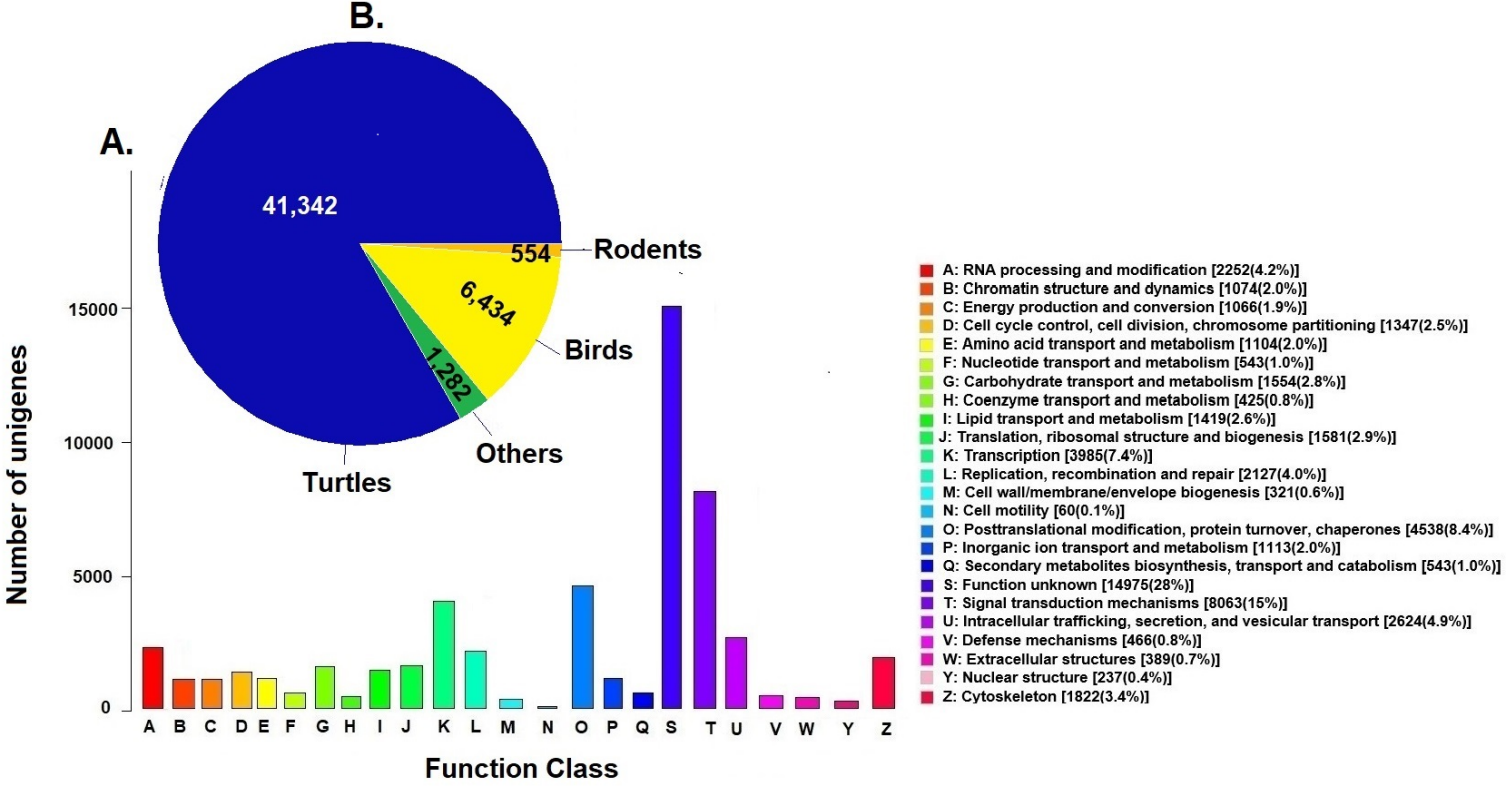
Clusters of Orthologous Groups (COGs). (A) Distribution of Clusters of Orthologous Groups (COGs) in 24 categories of the loggerhead sea turtle, *Caretta caretta*, transcriptome are presented. (B) Number of COGs identified that matched the unigenes of *Caretta caretta* in different species.

Unigenes were compared against the COG database for functional annotation with 49,612 genes (29.9%) classified into 24 functional categories in Clusters of Orthologous Groups (COG) (Fig. 2A). The category of genes with unknown function was the most represented (14,975, 28%) followed by signal transduction mechanisms (8,063, 15%) and post-transcriptional modification chaperones (4,538, 8.4%). It is important to note that 83% of COGs were inferred from turtle sequences (Testudines) (Fig. 2B), corroborating the annotation performed of the loggerhead turtle against the proteomes of the green sea turtle, the western painted turtle, and the softshell turtle, presented above.

### Annotation to KEGG pathways

**Figure 3:**
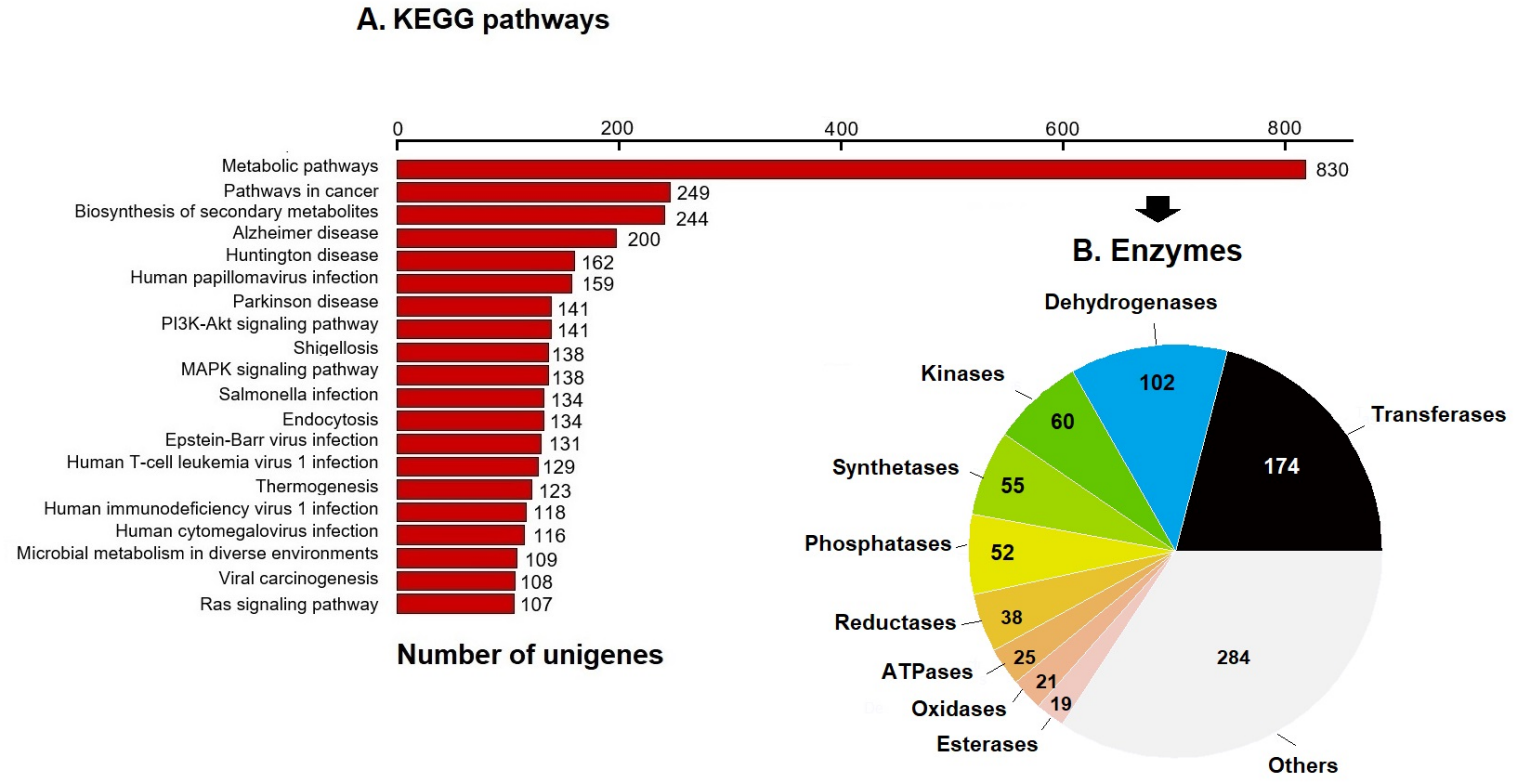
KEGG pathways and enzymes. (A) Top 20 KEGG pathways with the highest number of annotated unigenes of loggerhead sea turtle. (B) Enzymes identified in metabolic pathways are presented.

KEGG pathway analysis annotated 17,046 genes (10.28%) within 403 pathways (Fig. 3). The six most representative pathways were: metabolic pathways (830), pathways in cancer (249), in the biosynthesis of secondary metabolites (244), Alzheimer’s disease (200), Huntington disease (162), and human papillomavirus infection (159) (Fig. 3A). In metabolic pathways, the 830 enzymes identified were grouped into 174 transferases, 102 dehydrogenases, 60 kinases, 55 synthetases, 52 phosphatases, 38 reductases, 25 ATPases, 21 oxidases, 19 esterases, and several others (Fig. 3B).

### Prediction and characterization of proteins related to antioxidant activity

We further annotated the 180 protein sequences related to antioxidant activity (Fig. 1D) with the Gene Ontology database (GO: 0016209). In this group, 28 putative peroxiredoxins (Prdxs) and 50 putative thioredoxin peroxidases (Tnxs) stand out. Six of these proteins (Prdx1, Prdx3, Prdx5, Prdx6, Txn and Txnip) were characterized in this study, being the Prdxs and Tnxs, among the most studied antioxidant proteins in mammals (Nelson et al., 2018; Kang et al., 1998; Hwang et al., 2014).

The six characterized putative proteins were annotated in each of the five databases used

(Table S2), as well as in the annotation made against the proteomes of the green sea turtle, the western painted turtle, and the softshell turtle. The predicted proteins presented sizes between 106 and 392 aa that are equivalent to the sizes in *Homo sapiens*, and some turtles such as the green sea turtle, the western painted turtle, and the softshell turtle (Table 3).

**Table 3:** Characteristics of the putative loggerhead turtle peroxyredoxins and thioredoxins identified in this study. Structural domains and active sites are presented.

**Protein in** ***Cc***	**ORF**	**Length (aa)**	**MW (KDa)**	**Domain organization**	**Active site motif**
Prdx1	0359947_Caretta.p1	199	22.2	AhpC-TSA y 1-cysPrx_C	GGLG y FF
Prdx3	0433542_Caretta.p1	282	30.43	AhpC-TSA y 1-cysPrx_C	GGLG y YF
Prdx5	0406313_Caretta.p2	217	22.7	Redoxin	SQL
Prdx6	0316557_Caretta.p1	224	25	AhpC-TSA y 1-cysPrx_C	GDSWG
Txn	0304941_Caretta.p2	106	12	Thioredoxin	CPGC y CSVKC
Txnip	0383176_Caretta.p1	392	43.6	Arrestin_N y Arrestin_C	PDAP, PPCY y PNTP

### Identification of motifs and functional domains of putative proteins Prdx1, Prdx3, Prdx5, Prdx6, Txn and Txnip

**Figure 4:**
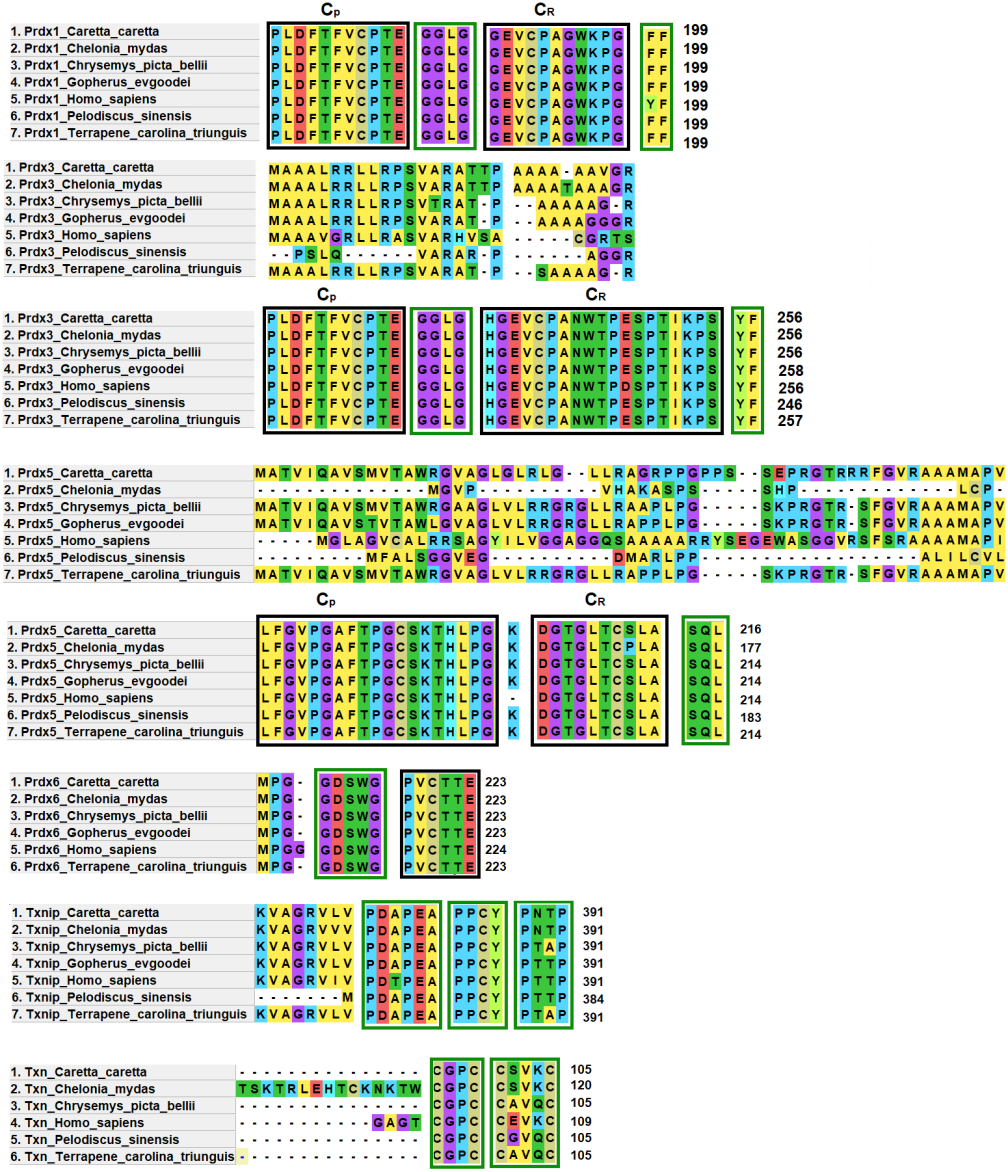
Structural motifs and catalitic centers of Prdxs, Txn and Txnip. Alignment of amino acid sequences of the proteins Prdx1, Prdx3, Prdx5, Prdx6, Txn, and Txnip reported in GenBank (Table S12) for the green sea turtle (*Chelonia mydas*), the western painted turtle (*Chrysemys picta bellii*), the soft-shell turtle (*Pelodiscus sinensis*), The three-toed box turtle (*Terrapene carolina triunguis*) and humans (*Homo sapiens*), and the one described in this study for loggerhead sea turtles (*Caretta caretta*). The boxes bordered with blue lines present the structural motifs, while boxes bordered with red lines present the catalytic centers in the species. CP and CR are conserved enzymatic regions. Alignment performed with Clustal Omega software (https://www.ebi.ac.uk/Tools/msa/clustalo/). (*) Represents identical amino acids and (. or:) represents amino acid similarity.

Prdx1, Prdx3 and Prdx5 have 2-Cys while Prdx6 has 1-Cys at the C_P_ and C_R_ active sites. These are typical characteristics of these enzymes (Nelson et al., 2018) (Fig. 4).

Prdx1, is a 199 aa protein with peroxisomal activity, and ability to form decamers and/or dodecamers (Nelson et al., 2018). In the analyzed turtle species, and in humans, it contained the motif “GGLG” and the conserved enzymatic regions C_P_ and C_R_ (Huang et al., 2017). These proteins contain active cysteine residues and lie within the two functional domains AhpC-TSA and 1-cysPrx_C. Tyrosine, together with phenylalanine, form the YF motif in mammalian species, which makes them susceptible to hyperoxidation (Huang et al., 2017; Bolduc et al., 2018). In the C-terminal region, the amino acid tyrosine has been replaced by phenylalanine (FF) in the green sea turtle, the western painted turtle, the soft-shell turtle and the loggerhead sea turtle (Fig. 4). It is possible that, in reptiles, this substitution confers greater resistance to hyperoxidation, and more resistance to bacteria, which lack both functional motifs (GGLG and YF) (Huang et al., 2017).

The Prdx3 protein, located within the mitochondria, has 282 aa. It frequently forms dodecamers (Cao, Bhella & Lindsay, 2007; Yewdall et al., 2016). The putative Prdx3 protein from loggerhead turtles shows the traditional GGLG and YF motifs that characterize Prdxs (Table 3), and that are conserved in the other turtles analyzed in this study (Fig. 4).

Prdx5 is a 217 amino acid long mitochondrial peroxiredoxin (L-Prdx5), also located in the cytosol, the peroxisome, and at times in the nucleus (Knoops, Goemaere & Van Der EV, 2011; Pirson, Clippe & Knoops, 2018). It is a monomer and additionally has peroxisomal activity (Hall, Karplus & Poole, 2009). It has a characteristic sequence at the N-terminus and a C-terminal SQL motif, which has been called peroxisome targeting sequence 1 (PTS1) and is highly conserved in all animal species (Table 3) (Knoops, Goemaere & Van Der EV, 2011; Kropotov et al., 2007). In the putative protein Prdx5 from loggerhead turtle, these motifs are preserved (Fig. 4).

The Prdx6 protein, located in the cytoplasm and lysosome (Kang et al., 1998), has a length of 224 aa. It is a bifunctional protein with peroxidase and phospholipase A2 activity (Manevich & Fisher, 2005). The putative protein Prdx6 from loggerhead turtle showed the conserved domains AhpC-TSA and 1-cysPrx_C typical of peroxiredoxins (Table 3). Within the AhpC-TSA domain, the DDSWG lipase motif and the PVCTTE catalytic center were found, which are conserved in most animal species (Liu et al., 2016).

The Txn protein is a 106 aa monomer located in the cytoplasm (Chen et al., 2002; Smeets et al., 2005). Txn shows a conserved CxxC motif at its active site that is related to an important role in cellular redox control and oxidative damage (Chen et al., 2002). It exhibits the typical CPGC and CSVKC structural motifs with some residue changes between turtles and humans (Fig. 4). The Txn protein is a member of the alpha-arrestin protein family that contains two characteristic domains, PxxP and PPxY which are known as binding motifs, and another active site identified as PNTP (Zhou & Chng, 2013; Hirata, Ito & Masutani, 2019; Patwari et al., 2006). These three basic structural motifs are seen in the putative Txn of loggerhead sea turtles (Table 3, Fig. 4). The putative protein in loggerhead sea turtle had a shorter length than that shown by Txn of the green sea turtle (120 aa) but equal to that of the western painted turtle, and *Homo sapiens* (106 aa) (Fig. 4). Structurally it is formed by a thioredoxin (Sc) domain. This same structure is presented by Txn1 from *Homo sapiens*, other mammals and birds (Parker, 2014; Weichsel et al., 1996).

Thioredoxin Txnip has 392 aa and is cytoplasmic. Txnip is a homodimer (Liyanage, Fernando & Lou, 2007). In loggerhead turtles, the Txnip sequence presented two conserved domains of arrestin and structural motifs (Fig. 4). Three of these motifs are highly conserved (PDAP, PPTY and PPCY) and were identified in the putative Txnip protein from loggerhead turtle and the other turtles analyzed in this study (Table 3). The fourth motif (PNTP) was common only in the Txnip identified in loggerhead turtle and green sea turtle; punctual changes were observed in the other species (Fig. 4).

### Phylogenetic relationship of Prdxs and Txns

**Figure 5:**
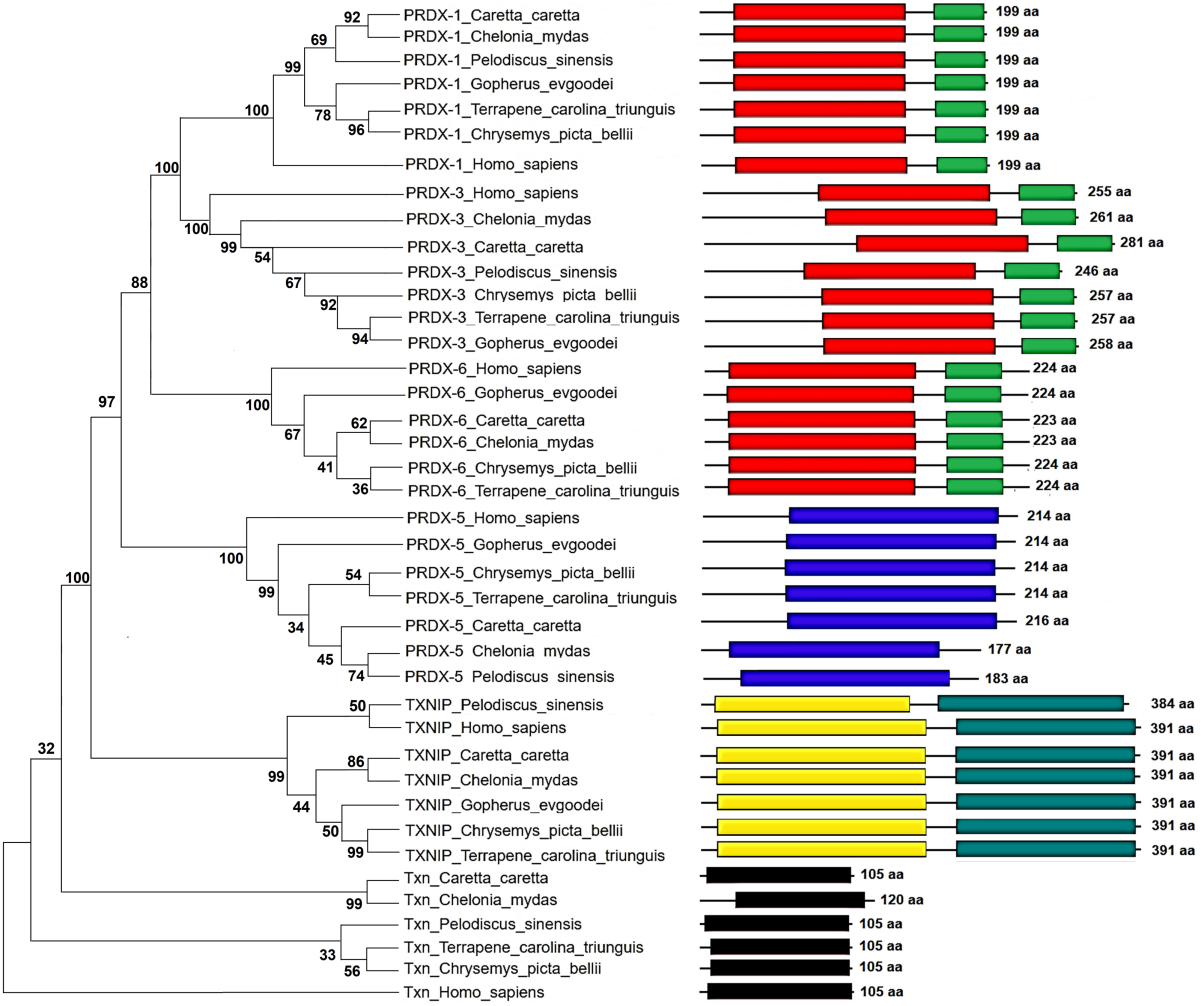
Phylogenetic relationship between Prdxs, Txn and Txnip. Phylogenetic tree showing the relationship between Prxd1, Prdx3, Prdx5, Prdx6, Txn y Txnip putative proteins and their structural domains in *Caretta caretta*, *Gopherus evgoodei*, *Terrapene carolina triunguis*, *Chrysemis picta belli*, *Pelodiscus sinensis* and *Chelonia mydas*. Humans (*Homo sapiens*) were used as external group. The phylogram was constructed on MEGA X using Maximum Parsimony method (MP). The percentage of replicate trees in which the associated taxa clustered together in the bootstrap test (1,000 replicates) are shown next to the branches.

The Maximum Parsimony tree and the relationship of the structural domains of the six proteins of loggerhead sea turtle compared to land turtles (*Gopherus evgoodei*), three-toed box turtle (*Terrapene carolina triunguis*), freshwater turtles (*Chrysemis picta belli* and *Pelodiscus sinensis*) and green turtle (*Chelonia mydas*) is presented in the Fig. 5. Humans (*Homo sapiens*) were included as an outgroup (Table S3). The consistency index was 0.926655 (0.922860), the retention index was 0.978069 (0.978069), and the composite index was 0.906332 (0.902621) for all sites and parsimony-informative sites (in parentheses) (Fig. 5). In some cases, the loggerhead sea turtle proteins are the most closely related to the *green sea turtle* proteins showing high bootstrap supports (between 86 and 99 for Prdx1, Prdx3, Txnip and Txn). This was expected, since these species are the most closely related in evolutionary terms (mean divergence time 48 MYA, Timetree) (Kumar et al., 2017). The Prdx1 and Prdx6 proteins show a very high conservation with structural domains located at the same sites and with similar length, 199 and 223–224 aa, respectively (the branches that relate these proteins show a bootstrap support of 100). The Prdx5, Txnip and Txn proteins are also conserved, but they present larger differences in protein length (Fig. 5). However, the structural motifs are preserved and the support for these branches is high (100, 99 and 32, respectively). Prdx3 is the protein that shows the greatest changes in length, with the structural motifs being maintained in all species and with a low bootstrap support for all the proteins in this branch (62) (Fig. 5, Table S3). Five of the homologous human proteins (Prdx1, Prdx3, Prdx6, Prdx5 and Txn) behaved as an external group, while Txnip was the only human protein that showed a close relationship with the homologous protein of *P. sinensis* with a medium bootstrap support (Fig. 5).

### Identification and evaluation of mutations of Prdxs, Txn and Txnip

Studying amino acid changes in coding regions is very important for analyzing protein function and how they are affected by mutations. To predict the effects of amino acid changes between loggerhead turtles and humans (*Homo sapiens*), we used the SIFT program (Ng & Henikoff, 2003). For the putative proteins Prdx5 and Txnip of the loggerhead turtle, 94 (43%) and 74 (19%) amino acid changes were found respectively, when compared with *Homo sapiens* (Table 4). However, for Prdx5, 55 (59%) of these amino acid changes were presented as tolerated and 18 as deleterious. Additionally, 11 insertions and 10 deletions were present. There were 32 tolerated changes in the Txnip protein and only 3 deleterious (Table 4). Predicting what the implications of these changes beyond the scope of this study.

The Prdx3 protein presented 52 (20%) changes in total, with 25 (48%) deleterious and 4 insertions. The Prdx1, Prdx6 and Txn proteins presented 18 (9%), 36 (16%) and 35 (33%) total changes, with 0, 3 and 2 deleterious changes, respectively (Table 4). It is important to note that the putative protein Prdx3 from loggerhead turtle showed an amino acid change in the C_R_ active site, tolerated (D84E change) (Ng & Henikoff, 2003). The putative Txnip protein exhibited an amino acid change in the PxxP structural motif, tolerated (T327A change) (Ng & Henikoff, 2003). Although the putative Prdx5 protein had the highest number of mutations (94, 10 deletions, and 11 insertions), none affected its C_P_ and C_R_ active sites, or the terminal SQL motif.

**Table 4:** Mutations identified in the peroxiredoxin and thioredoxin proteins predicted in *Caretta caretta* compared to those present in *Homo sapiens*.

**Protein**	**Mutations**	**Tolerant (%)**	**Intolerant (%)**	**Insertions (%)**	**Deletions (%)**
PRDX1	18	18 (100%)	0		
PRDX3	52	27 (52%)	25 (48%)	4 (26 aa )	
PRDX5	94	55 (58%)	18 (19%)	11 (12%)	10 (11%)
PRDX6	36	33 (92%)	3 (8%)		
TXN	35	33 (94%)	2 (6%)		
TXNIP	74	71 (9%)	3 (4%)		

### Physicochemical properties of Prdxs, Txn and Txnip

To gain further insight into the observed differences, we analyzed the physicochemical properties of the Prdx3, Prdx5 and Txnip proteins in the loggerhead turtle and *Homo sapiens* using the ProtParam program (Garg et al., 2016). Pronounced changes are observed between the Prdx3 protein of the loggerhead turtle and that of *Homo sapiens* (Table 5). In loggerhead turtles, Prdx3 has a higher molecular weight, due to the 26 additional amino acids that it possesses, a strong reduction in the estimated median life (from 30 h in *Homo sapiens* to only 1 in the loggerhead turtle), a lower aliphatic index, and a very high instability index (47.5), making it an unstable protein (Table 5). The proteins Prdx5 and Txnip of the loggerhead turtle showed physicochemical properties similar to those of *Homo sapiens*. Loggerhead turtle Prdx5 was a stable protein with a median life of 30 h, similar to humans, but Txnip presented a stability index of 46.6, well above the corresponding protein in *Homo sapiens* (Table 5).

**Table 5:** Physicochemical and secondary structure characterization of *Caretta caretta* Prdx3, Prdx5 and Txnip oxidative stress proteins. The analysis was carried out with the ProtParam and SOPMA programs.

**Especies**	***Caretta caretta***			***Homo sapiens***		
**Proteins**	**Prdx3**	**Prdx5**	**Txnip**	**Prdx3**	**Prdx5**	**Txnip**
**Molecular weight (KDa)**	30.43	22.72	43.67	27.56	22.08	43.66
**Isoelectric point**	8.46	9.36	5.72	–	8.93	7.46
**Median life (hours)**	1	30	30	30	30	30
**Aliphatic index**	85.44	91.2	81.41	91.41	91.68	83.15
**Average hydropathy (GRAVY)**	0.011	0.075	−0.22	0.075	0.152	−0.232
**Unstability index**	47.49	32.17	46.6	30.49	31.51	37.9
**Stability**	Unstable	Stable	Unstable	Stable	Stable	Stable
**Secondary structure**						
**Alpha helix**	35.59	35.65	14.58	32.16	31.31	15.35
**Random coil**	43.06	37.5	53.96	42.35	33.64	51.41
**Extended strand**	14.59	21.76	25.83	19.22	25.23	26.6
**Beta turn**	6.76	5.09	5.63	6.27	9.81	6.65

### 2D and 3D structures in the putative Prdxs, Txn and Txnip proteins

The 2D and 3D structures of Prdx3 and Txnip proteins of the loggerhead sea turtle were predicted and compared to their counterparts in *Homo sapiens* (Fig. S4). For this task, the SOPMA program (Geourjon & Deléage, 1995) was used to determine the 2D structure, and CPHmodels-3.2 Server (Nielsen et al., 2010) to construct the 3D structure. The 3D structures of the Prdx3 and Txnip proteins were modeled by homology. As templates, CPHmodels-3.2 used the 3D structures of the A and C chains of the Prdx3 (5JCG) and Txnip (4LL4) of *Homo sapiens*. The 3D structures predicted for loggerhead turtle Prdx3 and Txnip presented identity percentages of 94.8 and 75.3%, and coverage of 74.2 and 74.7% respectively against the template structures. The 3D structures of the predicted proteins were visualized with UCSF Chimera, a program for the interactive visualization and analysis of molecular structures. It was observed in both cases that Prdx3 and Txnip presented the same number of alpha helices and folded beta as those observed in *Homo sapiens* (Fig. S4). The Prdx3 of both loggerhead turtle and humans showed six alpha helices and seven folded beta sheets, while the Txnip showed 15 folded beta sheets (Figs. S4A, S4B, left and right).

## Discussion

The loggerhead sea turtle transcriptome was assembled using eight libraries obtained from the complete blood RNA. Subsequently, functional analyzes were performed and proteins related to oxidative stress were identified and characterized: four peroxiredoxins and two thioredoxins. This study presents the first characterization of the entire loggerhead sea turtle blood transcriptome using an Illumina paired-end sequencing strategy.

We obtained a high number of contigs in this *de novo* assembly but this is not unexpected in this type of assembly, including partial transcripts, intronic regions, chimeras, repetitions, and gene variants. As a result, the number of initially assembled transcripts greatly exceeds the number of complete protein coding genes (Montero-Mendieta et al., 2017; Moreno-Santillán et al., 2019). Bentley et al. (2017) previously obtained a transcriptome with 382,294 transcripts from six loggerhead sea turtles, which is in the same order of magnitude to the number of contigs in this study.

We also identified numerous protein kinase domains as expected. These enzymes play an important role in the regulation of intracellular metabolism, gene expression, cell signaling, protein regulation, cell transport, secretory processes, and integral actions in many areas, including growth and development, disease, apoptosis, and cellular responses to external stress (Rider et al., 2009). The protein kinase domain is particularly important in turtles because it plays a key role in freezing tolerance, possibly by transcribing antioxidant genes (Montero-Mendieta et al., 2017). Greenway & Storey (2000) subjected adult *Trachemys scripta* tortoises to 5 h of anoxia and found that two protein kinases (ERK and JNK) were upregulated in liver, heart, kidney, brain, and spleen. These results for an anoxia-tolerant animal suggest the potential importance of MAPKs in mediating adaptive responses to oxygen deprivation.

Another identified domain, the zinc finger C_2_H_2_, was also observed in high numbers, like those found in the Chinese three-keeled pond turtle (*Mauremys reevesii*), in which 1,943 domains of zinc finger C_2_H_2_ were identified (Yin et al., 2016). This motif is one of the largest and most prevalent in the family of proteins for specific binding to DNA sequences (Yin et al., 2016).

Zinc fingers represent one of the most common domains found in higher eukaryotic transcription factors (TFs) (Fedotova et al., 2017). Studies have shown that exposure to heavy metals, including mercury, generates the disruption of proteins that contain zinc fingers, which in turn induces structural and functional changes in proteins, generates cellular deterioration, alters the expression of the genes, signal transduction and DNA repair (Zawia et al., 2000; Crespo-López et al., 2009). *In vitro* experiments have identified that some zinc-finger-like TFs are affected by oxidative stress generated by nitric oxide (NO⋅), hydrogen peroxide (H_2_O_2_), singlet oxygen (O_2_), peroxide radicals (ROO⋅), and peroxynitrite (ONOO). Some of the changes may be reversible and others irreversible. Cells are capable of reversing the changes produced by nitrosative stress, but not those caused by oxidative stress (Kröncke et al., 2002). The conservation and evolution of this domain should be further studied in turtles.

Our results were similar to Wang et al. (2013) in the transcriptome of the *Pelodiscus sinensis* where the most representative category of orthologs was also genes with unknown function, with approximately 4,800 (14.6%) annotated orthologous genes. These data demonstrate the limited knowledge of the genetics of loggerhead sea turtles.

We also identified 8,063 orthologous genes related to signal transduction (T) and 4,538 with posttranslational modification (O), approximately 2,500 (T) and 2,000 (O) genes more than those obtained in the transcriptome of a hybrid of *P. sinensis* (Zhang et al., 2018). This could indicate that loggerhead turtles could present a different response in the regulation of cellular immune responses, compared to other turtle species such as *P. sinensis* (Liu et al., 2020), by expressing a greater number of genes related to signal transduction. Also, an explanation for these large differences in the expression of these genes may be the low depth of the transcriptome obtained from *P. sinensis* or the small sample size of that study. Additionally, the functional and structural differences of the peroxyredoxin enzymes should also be studied further in this underrepresented taxonomic group. These enzymes help regulate oxidative stress, participate in signal transduction, and modulate the immune response (Zhang et al., 2019), while also being key to understanding how the physiological responses to adverse events differ between sea and fresh water turtles.

In the *P. sinensis* turtle, a greater number of transcripts (26,496) were assigned to 242 KEEG pathways (Wang et al., 2013), which is well below the number of pathways assigned in the loggerhead turtle (403), showing a greater variety of metabolic events carried out in this sea turtle. It is important to note that the *P. sinensis* transcriptome was obtained from six different types of tissues, while that of the loggerhead turtle, in this study, was obtained from three developmental stages, which could explain the greater number of observed KEEG pathways.

### Peroxiredoxins and tioredoxins

In loggerhead sea turtles there is currently no knowledge of Prdxs and Trxs activity, thus these findings motivate the exploration of the molecular mechanisms related to their function. In this research, we identified 28 putative peroxiredoxins (Prdxs) and 50 putative thioredoxin peroxidases (Tnxs). Among these, Prdx1, Prdx3, Prdx5, and Prdx6 were chosen for study, according to the number and homology of the cysteines. Prdxs are classified into three classes: 1-Cys Prdx (Prdx6), 2-Cys Prdx (Prdx1, Prdx3) and atypical 2-Cys Prdx (Prdx5) (Zhang et al., 2019). Prdxs of all three classes were evaluated with both thioredoxins. From elsewhere, Trxs are considered proteins with anti-oxidant, anti-apoptotic and anti-inflammatory effects (Watanabe et al., 2010; Zhou et al., 2010; Zhou & Chng, 2013).

The putative proteins encoded by the Prx1, Prdx3, Prdx5, Prdx6, Txn, and Txnip genes were characterized in silico. In the sequence of the putative protein Prdx1, we identified the YF motif (Tyrosine-phenylalanine amino acids) which produces a susceptibility to hyperoxidation in mammals. In the green sea turtle, the western painted turtle, the soft-shell turtle, and the loggerhead sea turtle, the amino acid tyrosine has been replaced by phenylalanine forming the FF motive. It is possible that, in reptiles, this substitution confers resistance to hyperoxidation. This hypothesis should be studied thoroughly. We have identified a fourth motif (PNTP) in the Txnip putative protein in loggerhead and green sea turtles. In a study by Kim et al. (2015), differences in Txnip were reported in this same motif in fish and mammals. In fish, the motif was PLTP and in mammals PTTP. Analyzing these changes in the Txnip sequence, it can be concluded that this protein in sea turtles, loggerhead and green, is different from that of land turtles and freshwater turtles. This hypothesis should be evaluated by carrying out functional experiments that verify the antioxidant capacity of Txnip.

As expected, the six putative proteins studied for the loggerhead sea turtle have a closer phylogenetic relationship with those of the green sea turtle. When compared with the human homologous protein, the Prdx3 putative protein of the loggerhead turtle presented 25 deleterious mutations, four insertions, 26 additional amino acids, and a high reduction in the estimated median life, a lower aliphatic index, and a very high instability index. These factors make it an unstable protein similar to the Txnip protein.

Prdx3 in loggerhead turtles, when compared with *Homo sapiens*, contains a higher proportion of alpha helix, beta turn, and random coil. Txnip in loggerhead turtles shows a higher proportion of random coil, and a lower proportion of alpha helix and beta turn, when compared with *Homo sapiens* (Table 4). Both show a lower proportion of extended strand than *Homo sapiens* (Table 4). Observing 2D structures (Fig. S4), it is easy to see the variation between proteins, more pronounced in the Prdx3 protein than in Txnip, contrary to what is observed in the 3D structure where the most notable differences are seen in the Txnip protein.

According to our analysis, the Prdx protein is unstable, however, we have to consider that Prdx3 is a dodecamer, which may add some stability (Guruprasad, Reddy & Pandit, 1990). In vivo, the stability of the protein will depend not only on the intrinsic nature of the protein but also on the conditions of the protein medium (Gamage et al., 2019). Traditional 2-Cys Prdxs can alternate between dimers and decameric or dodecameric rings during their catalytic cycle. This conformational change to a quaternary protein is not fully understood (Yewdall et al., 2018).

## Conclusions

In this study, we have obtained for the first time the complete blood transcriptome of loggerhead sea turtles and annotated the sequences of 51,798 protein-coding transcripts that represent only 32% of the identified unigenes. In this way, 68% of the unigenes did not match against any known protein to date. This fact represents an important challenge for the understanding of the loggerhead sea turtles’ genetics. Moreover, the largest number of protein domains identified in this study are related to protein kinase domains, which regulate tolerance to anoxia and, therefore, the decrease in metabolism. This issue has received much attention in past decades, but its explanation is not clear yet. The functions of the largest number of orthologous genes identified are also unknown, which represents an important field for future study. KEGG pathway analysis annotated only 17,046 genes (10.28%) within 403 pathways leaving more than 80% of genes unidentified. However, compared with previous studies of turtles such as P. sinensis, we succeeded at identifying almost double the number of metabolic pathways. This quantity of identified pathways is perhaps due to the use of samples from hatchlings, juveniles and adults of the loggerhead sea turtle. A study about differential expression genes could clarify the number of metabolic pathways found in this study.

In the sequence of the putative protein Prdx1 we identified the YF motif which produces a susceptibility to hyperoxidation in mammals. In loggerhead sea turtles, we found that this motive changed (FF), and hypothesize that this substitution confers resistance to hyperoxidation. We have identified a fourth motif (PNTP) in the Txnip putative protein in loggerhead and green sea turtles. This protein is different from that of land turtles and freshwater turtles, but its function remains unknown.

The six putative proteins studied for the loggerhead sea turtle have a closer phylogenetic relationship with those of the green sea turtle. The protein Prdx3, putative protein of the loggerhead turtle, presented the most changes when compared with the human homologous protein. This unstable protein presented a shorter mean life, however, we observed high similarity between the 3D structures of human and loggerhead turtle Prdx3. The loggerhead sea turtle transcriptome we obtained and analyzed in this study provides a comprehensive reference for further molecular studies of this endangered species.

## Supplemental Information

10.7717/peerj.12395/supp-1 Supplemental Information 1 Sequences of Prdx1, Prdx3, Prdx5, Prdx6, Txn and Txnip used in this study for the alignment and phylogenetic analysis

10.7717/peerj.12395/supp-2 Supplemental Information 2 Supplementary Tables

10.7717/peerj.12395/supp-3 Supplemental Information 3 Supplemetary Figures

## Additional Information and Declarations

### Field Study Permissions

The following information was supplied relating to field study approvals (i.e., approving body and any reference numbers): Samples were obtained under a research permit that was granted by the Ministry of Environment and Territorial Development (# 24 of June 22, 2012) and Contract for Access to Genetic Resources (# 64 of 129 April 23, 2013). A permit for the collection of samples of Colombian biodiversity, issued to the Jorge Tadeo Lozano University, was obtained for this study (Resolution 1271 of October 23, 2014, IDB040I File).

### Data Availability

The following information was supplied regarding data availability: The RNA sequencing reads are available at SRA NCBI: BioProject PRJNA560561, Biosample: SAMN06350885, SAMN12591161, SAMN12591162, SAMN12591163, SAMN12591164, SAMN12591165, SAMN12591166, SAMN12591173 Sequence Read Archive: SRR10032986, SRR10032987, SRR10032988, SRR10032989, SRR10032990, SRR10032991, SRR10032992, SRR5330501, SRR10032986, SRR10032987, SRR10032988, SRR10032989, SRR10032990, SRR10032991, SRR10032992 and SRR5330501. The transcriptome assembly is available at TSA: GIBB00000000. The version described in this article is the first version. The sequences of genes and proteins predicted in this study are available at GenBank: MT833868 (Prdx1), MT833869 (Prdx3), MT833870 (Prdx5), MT833871 (Prdx6), MT833872 (Txn), and MT833873 (Txnip).
